# Improving posture-motor dual-task with a supraposture-focus strategy in young and elderly adults

**DOI:** 10.1371/journal.pone.0170687

**Published:** 2017-02-02

**Authors:** Shu-Han Yu, Cheng-Ya Huang

**Affiliations:** 1 Physical Therapy Center, National Taiwan University Hospital, Taipei City, Taiwan; 2 School and Graduate Institute of Physical Therapy, College of Medicine, National Taiwan University, Taipei City, Taiwan; Tokai University, JAPAN

## Abstract

In a postural-suprapostural task, appropriate prioritization is necessary to achieve task goals and maintain postural stability. A “posture-first” principle is typically favored by elderly people in order to secure stance stability, but this comes at the cost of reduced suprapostural performance. Using a postural-suprapostural task with a motor suprapostural goal, this study investigated differences between young and older adults in dual-task cost across varying task prioritization paradigms. Eighteen healthy young (mean age: 24.8 ± 5.2 years) and 18 older (mean age: 68.8 ± 3.7 years) adults executed a designated force-matching task from a stabilometer board using either a stabilometer stance (posture-focus strategy) or force-matching (supraposture-focus strategy) as the primary task. The dual-task effect (DTE: % change in dual-task condition; positive value: dual-task benefit, negative value: dual-task cost) of force-matching error and reaction time (RT), posture error, and approximate entropy (ApEn) of stabilometer movement were measured. When using the supraposture-focus strategy, young adults exhibited larger DTE values in each behavioral parameter than when using the posture-focus strategy. The older adults using the supraposture-focus strategy also attained larger DTE values for posture error, stabilometer movement ApEn, and force-matching error than when using the posture-focus strategy. These results suggest that the supraposture-focus strategy exerted an increased dual-task benefit for posture-motor dual-tasking in both healthy young and elderly adults. The present findings imply that the older adults should make use of the supraposture-focus strategy for fall prevention during dual-task execution.

## Introduction

Upright stance is a daily task that requires minimal attentional resources. A secondary task (suprapostural task) increases the demand on attentional resources especially in geriatric or neurologically impaired populations [1,2]. When undertaking a postural-suprapostural dual-task, one can prioritize either the postural or suprapostural task; each strategy results in different task outcomes and resource allocations. Young adults focusing on postural task often deteriorate automatic control of posture, resulting in increased postural instability, and postural stability increased when withdrawing attention from the postural task [3–6]. Aging causes degeneration of sensorimotor systems required for stance stability [7,8], and reduces attentional resource availability for undertaking dual-task conditions [1,9]. Therefore, older adults typically prioritize the postural task as a means of fall prevention [10–12]. It appears that under dual-task conditions without imposed task-prioritization, the unconscious “posture-first” strategy is an appropriate solution for the age-related postural destabilization occurring in older adults, as they are less able than young adults to reduce postural sway by adopting automatic postural control in the “supraposture-first” strategy [13–16].

However, regarding to impose task prioritization in a postural-suprapostural task, the optimal postural strategy for older adults is still an issue of debate. A few studies have reported a decrease in postural stability when older people performed a postural-suprapostural task with paying attention to the suprapostural tasks [17,18], whereas other studies have shown enhanced postural stability when more attention was directed to the suprapostural task [19,20]. In fact, the dual-task cost increased proportionally with the task compatibility of two concurrent tasks, especially in older adults [21,22] who showed a larger performance decrement in posture-motor dual-tasking than posture-cognition dual-tasking [23]. Moreover, variations in task prioritization effect could be ascribed to the nature of the instructions given in the experiment [18,24] as the instruction format was found to influence relative resource allocation between the two tasks. Verbal instruction affects the degree of attention being devoted to the secondary task. In the extreme case, subjects may abort the suprapostural task for the sake of the postural task.

In light of this inconsistency in findings of a task prioritization effect on postural control in older adults, the purpose of this study was to reexamine the influence of task prioritization (posture-focus (PF) versus supraposture-focus (SF)) in both young and older adults who were given specific task-priority instructions to maintain postural balance while performing a motor suprapostural task simultaneously. In the present study, a precision grip task was used as the motor suprapostural task. Besides fine force control, a precision grip task required cognitive aspects of motor control such as response to an executive signal as fast as possible and sensorimotor integration during movement preparation period [25,26]. With this complex posture-motor-cognitive task, sophisticated allocation of attentional resources is needed by intensive resource competition between the postural and suprapostural tasks and/or by simultaneously coordinating the two tasks, pertaining to the relative importance of resource allocation between the postural and suprapostural tasks when task prioritization varies. Our main hypotheses were that (1) postural and motor performance of a postural-suprapostural task would be differently affected by the strategy of task prioritization and (2) the effects of task prioritization would be different between young and older adults.

## Materials and methods

### Ethical approval

The experiment was conducted in accordance with the Declaration of Helsinki and with the approval of the local ethics committee (National Taiwan University Hospital Research Ethics Committee; no. 201312077RINC). Written informed consent was obtained from all participants.

### Participants

Eighteen healthy young adults (8 male, 10 female; age range: 20.0–33.6 years; mean age: 24.8 ± 5.2 years) and eighteen healthy older adults (7 male, 11 female; age range: 61.8–77.0 years; mean age: 68.8 ± 3.7 years) were recruited in the study. All subjects were right-handed with normal or corrected-to-normal vision and were included on the basis of the following criteria: no previous physical, neurological, or sensory disorders, no medication that might influence their balance or cognition, and no history of fall in the previous 12 months. In addition, older adults were excluded if Mini Mental State Examination (MMSE) scores were less than 24 points.

### Experimental apparatus

**Fig 1:**
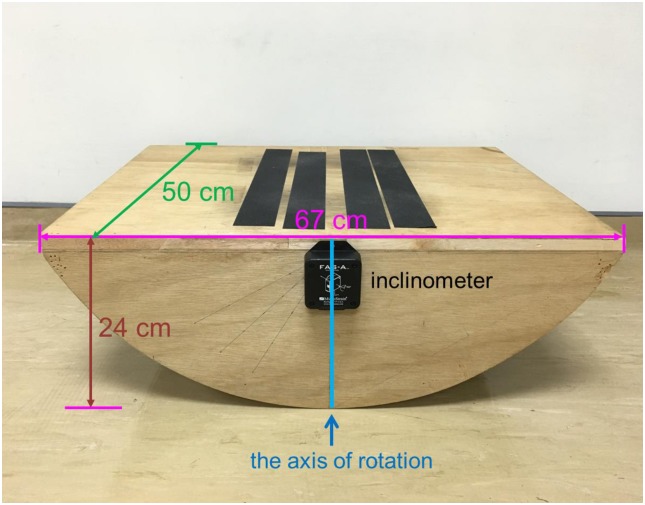
The stabilometer setup.

**Fig 2:**
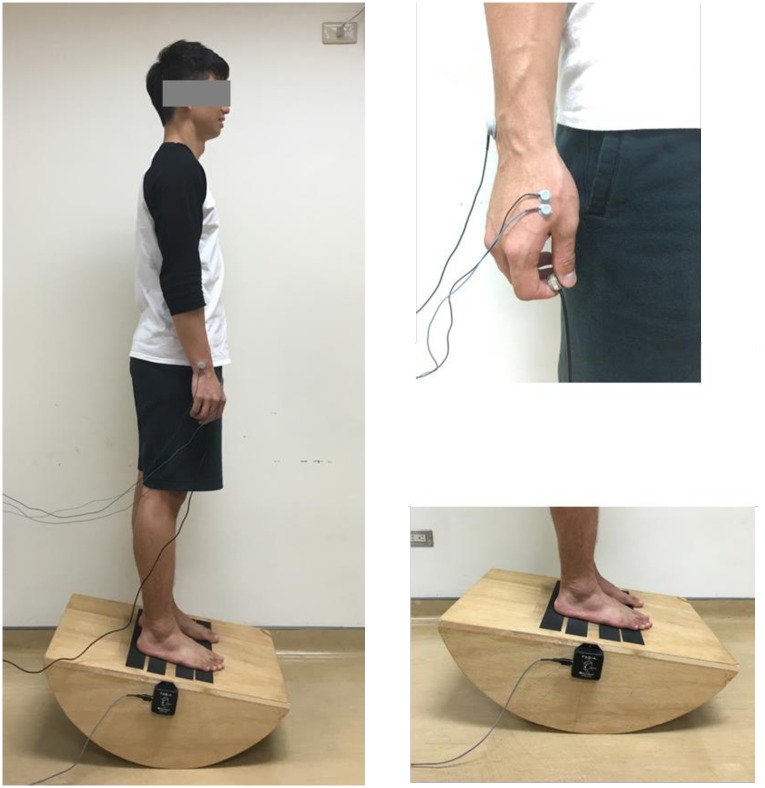
The standard position of participants in the experiment.

Participants were requested to perform a force-matching precision grip task (suprapostural task) while standing on a stabilometer (postural task; 67-cm length × 50-cm width × 24-cm height, anterior or posterior tilting angle: 50 degrees for each direction)(Fig 1) while adopting either PF or SF strategy. The standard position of stabilometer stance was that the participants stood with both heels mediolaterally parallel, separated by a distance equivalent to the individual’s shoulder width. When the stabilometer was kept in horizontal, the axis of rotation was just passed through the anterior aspect of the participant’s bilateral lateral malleolus. We marked the positions of each participant’s feet on the stabilomter to make sure the positions of the feet were identical throughout the study. The standard position of the upper extremities was that the participants hung their arms by the sides of the trunk in a relaxed manner and held the load cell by their thumb and index finger of right hand (Fig 2).

For the postural task, participants were asked to maintain their balance on the stabilometer at a target angle. An inclinometer (Model: FAS-A, MicroStrain, USA) was mounted on the center of the stabilometer plate to measure the tilting angle of the stabilometer. Before the main experiment, each participant was asked to tilt the stabilometer to its maximal anterior tilt by plantarflexing his/her ankle joints. The value of maximal tilting angle was recorded when the participant could maintain his/her balance in this maximal tilting angle without bending trunk for 5 seconds. Then, the postural target was set as 50% of the maximal anterior tilting angle. For the suprapostural task, participants executed a force-matching task with their right index finger and thumb, and the level of force output was recorded by a load cell (15-mm diameter × 10-mm thickness, net weight = 7 grams; Model: LCS, Nippon Tokushu Sokki Co., Japan). The load cell was connected to a distribution box via a thin and flexible wire so that the force apparatus would not act as a stable support in stance by providing a mechanical effect. Maximum voluntary contraction (MVC) of precision grip was recorded before the experiment and 50% of the MVC force was set as the target force. The participants needed to perform a quick precision grip (force impulse duration < 0.8 second) to couple the peak precision force with the target force when receiving executive auditory cues from speakers. The auditory cues consisted of fifteen warning-executive signal pairs (warning: 800 Hz, 100 ms; executive: 500Hz, 100 ms) in an 80-second trial. Because the first three warning-executive pairs in each trial were used for task preparation and not included in data analysis, a 1-second interval between signal pairs was set for the first three warning-executive pairs. For the 4^th^ to 15^th^ warning-executive pairs, a warning tone was randomly presented at intervals of 1.5, 1.8, 2.1, 2.4, 2.7, or 3.0 seconds before an executive tone for minimizing prediction of force-matching execution, and each interval was presented twice. The interval between the end of the executive tone and the beginning of the next warning tone was 3.5 seconds.

The auditory cues and target signals for the force-matching and the postural tasks were generated using the LabVIEW software (National Instrument, Austin, TX, USA). Both target signals were displayed on a 22-inch monitor positioned at eye-level, 60 cm from the participant’s head. By separating scale-tuning the vertical axis of the display of force output and stabilometer tilting angle in the LabVIEW platform, both target signals of force-matching and posture could be displayed in the same position of the monitor to reduce the visual load during the experiment. In addition, the activation of the first dorsal interosseous (FDI) muscle was monitored with surface electromyogram (EMG) in a bipolar arrangement (Ag/AgCl, 1.1 cm in diameter, Model: F-E9M-40-5, GRASS) and an AC amplifier (gain: 5000, cut-off frequency: 1 and 300 Hz; Model: QP511, GRASS, USA). All behavioral signals, including stabilometer movement, precision grip force, and EMG of FDI muscle, were synchronized with a sampling rate of 1 kHz.

### Procedures

There were six experimental conditions in this study (Table 1), comprising two postural-suprapostural dual-tasks with PF and SF strategies (D_PF and D_SF conditions), two single force-matching tasks (S_FNF and S_FWF conditions), and two single stabilometer standing postural tasks (S_PWF and S_PNF conditions). In the S_FWF and S_FNF conditions, the participants executed the force-matching task with or without visual feedback from gripping force while standing on a static wooden box (67 cm × 50 cm × 24 cm) of the same size as the stabilometer. In the S_PWF and S_PNF conditions, the participants held the load cell without executing the force-matching task and maintained their balance in the target position with or without visual feedback from stabilometer tilting angle.

**Table 1:** Experimental conditions.

Condition	Postural task	Suprapostural task
*Dual task*		
D_PF	Stabilometer standing (with performance feedback)	Force-matching (no performance feedback)
D_SF	Stabilometer standing (no performance feedback)	Force-matching (with performance feedback)
*Single postural task*		
S_PWF	Stabilometer standing (with performance feedback)	No force-matching
S_PNF	Stabilometer standing (no performance feedback)	No force-matching
*Single force-matching task*		
S_FWF	Quiet standing	Force-matching (with performance feedback)
S_FNF	Quiet standing	Force-matching (no performance feedback)

S_PWF and S_FNF were the single control conditions for the D_PF condition.

S_PNF and S_FWF were the single control conditions for the D_SF condition.

**Fig 3:**
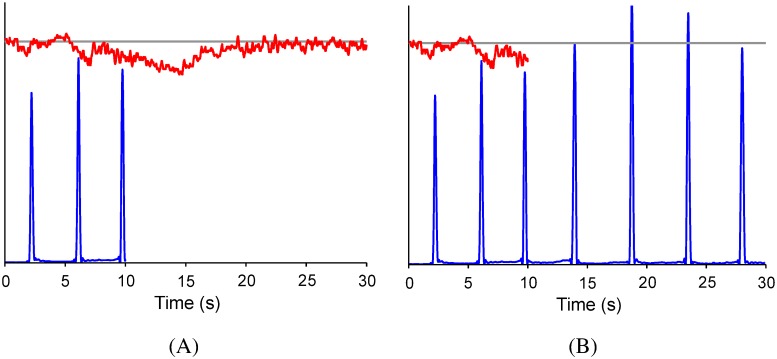
Sample recordings of the target, exerted force and stabilometer movement in two postural-suprapostural dual tasks. The gray line represents the postural target and force-matching target, the blue line represents the exerted force and the red line represents the stabilometer movement. (A) D_PF condition and (B) D_SF condition.

In the two dual-task conditions, specific instructions for task prioritization were given to prompt resource allocation by employing an optimum-maximum method [22,27,28]. In the optimum-maximum method, the task requirement would be specifically instructed for both primary and secondary tasks. In addition, visual information of both force output and stabilometer movement was used to reinforce the attention devoted to the high-priority task. In the D_PF condition, both postural and force-matching performance were showed on the monitor for the first 10 seconds (the first 10 seconds included the first 3 force-matching actions), and then the force-matching performance display was removed. The participants were instructed as follows: “the postural task is the primary task, maintaining the stabilometer tilting angle as closely as possible to the target angle, and then to perform your best on the force-matching task.” In the D_SF condition, both postural and force-matching performances were shown on the monitor for the first 10 seconds, and then the postural performance display was removed. The participants were instructed as follows: “the force-matching task is the primary task, generating the force peak of each force-matching action as quickly and as closely as possible to the target force and then to perform your best on the postural task.” Fig 3(A) and 3(B) provide representative examples of target and task performance in the D_PF and D_SF conditions. Because performance feedback was provided depending on the task prioritization in the dual-task conditions, participants had to perform single postural and force-matching tasks with or without performance feedback. As in the dual-task conditions, performance feedback of force output in the S_PWF condition and stabilometer movement in the S_FWF condition were provided for the first 10 seconds in each trial and then removed from the monitor. There were five trials for each experimental condition and each participant was tested in a random order across experimental conditions.

### Data analysis

For postural performance, the inclinometer data was conditioned with a zero phase low-pass filter (cut-off frequency: 6 Hz), followed by linear transformation to degree out of balance (1 mv output of inclinometer = 90 degree inclination). The inclinometer data from the 11^th^ to 80^th^ seconds was selected for calculation of angle error, and approximate entropy (ApEn) of the postural task. The dependent variable used to assess posture error was a root mean square error (RMSE), which was calculated as follows:

$$\text{RMSE}=\sqrt{\frac{1}{\text{N}}\sum_{\text{i}=1}^{\text{N}}{\left({\text{x}}_{\text{i}}-\text{t}\right)}^{2}}$$

where N is the number of inclinometer data points, t is the target angle, and x_i_ is the time-series tilting angle derived from the inclinometer data. For approximate entropy (ApEn) calculation, the trajectories of stabilometer tilting angle was first normalized with the standard deviation of the time series. ApEn is mathematically formulated as:

$$ApEn(m,r)=\text{log}\left[\frac{C_{m}\left(r\right)}{C_{m+1}\left(r\right)}\right]$$

The algorithm obtains the average count of the recurrence of vectors of length m and m + 1 within a tolerance range of r for given unit-variance normalized sway data [29]. If a completely predictable time-series with high regularity, values of C_m_(r) will be very close to C_m+1_(r), yielding a log-probability (ApEn) of zero. The value of ApEn was between 0 and 2, which is positively correlated with the amount of attention allocated to postural control. An ApEn value closer to 0 indicates greater postural regularity and more attentional resources devoted to postural control, while a value near 2 represents higher postural irregularity [30,31]. For suprapostural performance, the force error was defined as |*PPF*−*TF*| (PPF: peak precision-grip force, TF: target force) from the 4^th^ to 15^th^ force-matching actions in each trial. The FDI EMG onset was determined as the first point in a time frame from which the next 50 ms of the EMG activity exceeded the 2 times standard deviations of the resting EMG. The reaction time (RT), the time difference between executive tone and FDI EMG onset, was determined to represent the duration of central processing for executing the force-matching task [32]. Comparing to RT estimated by the time interval between the executive signal and the onset of the force or finger movement, the RT estimated between executive tone and EMG onset of the prime mover muscle is more reliable with potentially excluding electromechanical delay variability among participants [33–35].

To measure the effect of task prioritization on dual-task performance, the dual-task effect (DTE) of each outcome measure was calculated separately for PF and SF strategies. DTE represents the relative change on dual-task performance compared to single-task performance with a positive value indicating dual-task benefit and a negative value indicating dual-task cost. The posture error DTE was calculated with the following equation:

$$Posture\;error\;DTE_{PF}(\%)=-\frac{D\_PF-S\_PWF}{S\_PWF}\times 100\%,$$

or

$$Posture\;error\;DTE_{SF}(\%)=-\frac{D\_SF-S\_PNF}{S\_PNF}\times 100\%.$$

Since an increase in posture ApEn represents more automatic postural control [30,31], the posture ApEn DTE was calculated as follow equation:

$$Posture\;ApEn\;DTE_{PF}(\%)=\frac{D\_PF-S\_PWF}{S\_PWF}\times 100\%,$$

or

$$Posture\;ApEn\;DTE_{SF}(\%)=\frac{D\_SF-S\_PNF}{S\_PNF}\times 100\%.$$

the force error DTE was formulated with the following equation:

$$Force\;error\;DTE_{PF}(\%)=-\frac{D\_PF-S\_FNF}{S\_FNF}\times 100\%,$$

or

$$Force\;error\;DTE_{SF}(\%)=-\frac{D\_SF-S\_FWF}{S\_FWF}\times 100\%.$$

and the force RT DTE was formulated with the following equation:

$$Force\;RT\;DTE_{PF}(\%)=-\frac{D\_PF-S\_FNF}{S\_FNF}\times 100\%,$$

or

$$Force\;RT\;DTE_{SF}(\%)=-\frac{D\_SF-S\_FWF}{S\_FWF}\times 100\%.$$

### Statistical analysis

The effects of task prioritization (PF vs. SF) and age (young vs. older) on absolute dual-task values and DTE values of posture error, posture ApEn, force error, and force RT were examined using a 2 × 2 mixed ANOVA. When necessary, post-hoc least significant difference (LSD) comparisons were performed. The level of significance was set at *p* < .05. Signal processing and statistical analysis was completed by using MatLab v. R2008a (Mathworks, Natick, MA, USA) and the statistical package for SPSS statistics v. 17.0 (SPSS Inc., Chicago, IL, USA). All data were presented were mean ± standard error.

## Results

Table 2 shows the baseline characteristics of subjects including the values of force-matching target, postural target and MMSE score. The young and older groups were similar in target force of the force-matching task and target angle of the postural task (p > 0.05). The MMSE score range was 25–30. For the older adult whose MMSE equal to 25 score, the years of education was eight years.

**Table 2:** Baseline characteristics of subjects.

Characteristic	Young adults (n = 18)	Older adults (n = 18)	t-test
Force-matching target (N)	2.03 ± 0.82	1.82 ± 0.48	*p* = 0.35
Postural target (degree)	10.76 ± 3.38	9.49 ± 1.58	*p* = 0.16
MMSE (point)	-	29.33 ± 1.28	-

Data are represented as mean ± standard deviation.

Tables 3 and 4 show the results for absolute measures of postural and suprapostural task performance under single-task condition and dual-task, respectively. In the dual-task conditions (Table 4), the ANOVA results suggested a significant age effect on absolute values of posture error and force RT (posture error: F_1,34_ = 30.27, *p* < .001; force RT: F_1,34_ = 19.13, *p* < .001), with larger posture error and force RT in the older group. On the other hand, instructions to task prioritization caused significant changes in suprapostural performance (force error: F_1,34_ = 36.32, *p* < .001; force RT: F_1,34_ = 25.10, *p* < .001) but not in postural performance (posture error: F_1,34_ = .002, *p* = .96; posture ApEn: F_1,34_ = 1.30, *p* = .26). Both young and older groups have smaller force error and force RT in the D_SF condition. It was worth to note that even posture feedback was eliminated in the D_SF condition, the posture error did not statistically increased in the D_SF condition, comparing to the D_PF condition.

**Table 3:** Absolute values of postural and suprapostural performance in the single-task conditions.

		single postural task		single force-matching task	
		*S_PWF*	*S_PNF*	*S_FWF*	*S_FNF*
Posture error (degree)	Young	0.89 ± 0.06	1.44 ± 0.13	-	-
	Older	1.75 ± 0.18	2.19 ± 0.15	-	-
Posture ApEn (×10^−2^)	Young	2.69 ± 0.17	2.61 ± 0.14	-	-
	Older	2.54 ± 0.15	2.57 ± 0.18	-	-
Force error (N)	Young	-	-	0.21 ± 0.02	0.45 ± 0.07
	Older	-	-	0.20 ± 0.01	0.38 ± 0.05
Force RT (ms)	Young	-	-	279.08 ± 11.50	308.27 ± 15.76
	Older	-	-	407.26 ± 17.69	425.77 ± 18.88

Data are represented as mean ± standard error.

**Table 4:** Absolute values of postural and suprapostural performance in the dual-task conditions.

		posture-focus	suprapostural-focus
		*D_PF*	*D_SF*
Posture error (degree)	Young	0.92 ± 0.07	1.01 ± 0.09
	Older	2.21 ± 0.23***	2.11 ± 0.22***
Posture ApEn (×10^−2^)	Young	2.73 ± 0.18	2.78 ± 0.14
	Older	2.37 ± 0.16	2.54 ± 0.18
Force error (N)	Young	0.47 ± 0.07	0.18 ± 0.02^††^
	Older	0.56 ± 0.09	0.21 ± 0.01^†††^
Force RT (ms)	Young	340.59 ± 20.05	285.33 ± 13.52^†††^
	Older	428.71 ± 17.79**	398.66 ± 17.45^†^^,^***

Data are represented as mean ± standard error.

^†^indicates a significant difference between PF and SF strategies (p < .05).

^††^indicates a significant difference between PF and SF strategies (p < .01).

^†††^indicates a significant difference between PF and SF strategies (p < .001).

**indicates a significant difference between young and older groups (p < .01).

***indicates a significant difference between young and older groups (p < .001).

**Fig 4:**
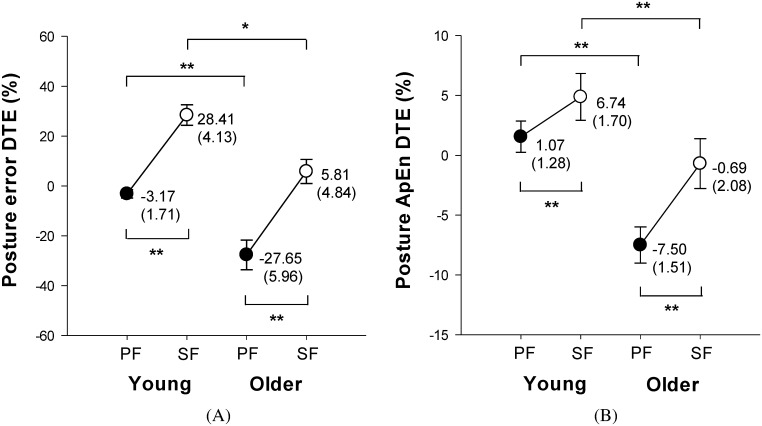
Effects of age and task prioritization on postural performance. (A) posture error DTE, and (B) posture ApEn DTE. *p < .05, **p < .01.

Fig 4(A) and 4(B) display the means and standard errors of DTEs for postural parameters. The ANOVA results suggested a significant age effect and task prioritization effect on DTEs of posture error (age: F_1,34_ = 22.89, *p* < .001; task prioritization: F_1,34_ = 69.36, *p* < .001) and posture ApEn (age: F_1,34_ = 16.94, *p* < .001; task prioritization: F_1,34_ = 21.51, *p* < .001). For the SF strategy parameter, large DTE values for posture error and posture ApEn were observed in both young and older adults. Most notably, posture error DTE polarized from negative to positive indicating SF strategy could result in dual-task benefit for postural stability. As for age effect, both posture error DTE and posture ApEn DTE were greater in young adults than in older adults, irrespective of task prioritization strategy.

**Fig 5:**
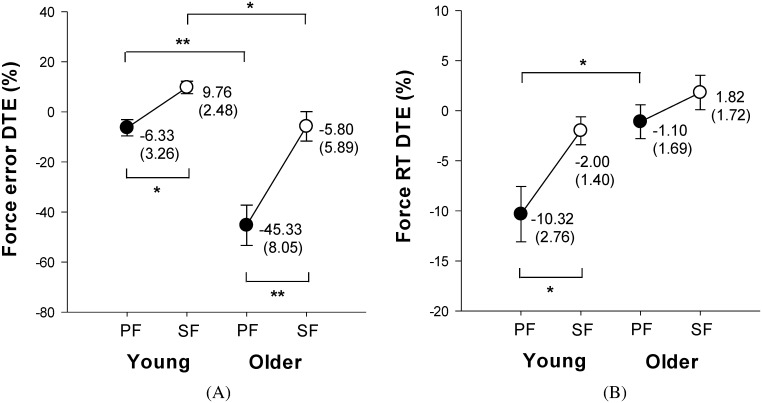
Effects of age and task prioritization on suprapostural performance. (A) force error DTE, and (B) force RT DTE. *p < .05, **p < .01.

Fig 5(A) and 5(B) display the means and standard errors of DTEs for suprapostural parameters. The ANOVA results suggested significant age (F_1,34_ = 26.15, *p* < .001) and task prioritization (F_1,34_ = 26.10, *p* < .001) effects on force error DTE. Similar to the posture error DTE, SF strategy resulted in larger force error DTE in both young and older adults, and force error DTE was larger in the young adults than that in the older adults. With respect to force RT DTE, ANOVA showed a significant age effect (F_1,34_ = 9.17, *p* < .01) and task prioritization effect (F_1,34_ = 10.30, *p* < .01) on force RT DTE. Post-hoc analysis revealed that force RT DTE was greater in the older adults than that in the young adults when employing a PF strategy. Moreover, we found that SF strategy caused larger force RT DTE in the young adults, but not in the older adults.

## Discussion

This study aimed to determine the relative contribution of task prioritization on DTEs between young and older adults in dual-task performance with a motor suprapostural goal. In instances where specific instruction were given for both the primary and secondary tasks, our results demonstrated that both postural and suprapostural performance benefited from an SF strategy in young and older adults. This suggests that SF is a better task prioritization strategy than PF for healthy adults, regardless of age.

Although an unconscious “posture-first principle” was supported in dual task conditions when no instruction about which task to prioritize was given [13–16], the finding of larger DTE values in posture error and force error from the present study shows that the “supraposture-focus (SF)” strategy was the better task prioritization strategy for older adults when performing a posture-motor-cognitive task. According to the adaptive resource-sharing framework for dual-tasking, a facilitatory pattern can be observed when more available resources are dedicated to the suprapostural task [36,37]. The precision of suprapostural performance can be aided by postural adjustments of sway reduction. On the other hand, the constrained action hypothesis suggests that focusing attention on a highly automated behavior, such as postural control, would constrain automaticity in the postural control process resulting in postural instability [2,3,38]. Although aging is correlated with declines in sensorimotor system performance and attentional resource availability [1,39], and all DTE parameters (except force RT) in older adults were smaller than those in young adults in our study, older adults still benefited from the SF strategy. Some studies reported that control strategies involved in upright stance are similar in young and older adults [40], but older adults may recruit additional neural resource to keep postural balance comparable to those obtained by young adults [41]. A compensatory age-related increase in brain resources to minimize dual-task interference has been reported previously; for instance, concurrent execution of visuomotor drawing and mental arithmetic counting has been reported [42]. In addition, the capacity for automatic processing of posture declines in older adults [43]. When using an SF strategy, older adults could facilitate force-matching accuracy by enhancing posture stability and automaticity, as in young adults (Figs 4 and 5(A)).

Another important finding of this study is that accuracy and RT of the force-matching task were differently affected by the PF strategy in the young and older groups. For the PF strategy, young adults showed more negative RT (-10.32 ± 2.76%) but less negative force-matching error (-6.33 ± 3.26%), whereas older adults showed a huge negativity of force-matching error (-45.33 ± 8.05%) but less negativity of RT (-1.10 ± 1.69%). Kelly et al. (2010) [44] reported that response latency in the suprapostural task was longer when the young subjects focused on the walking task than when they focused on the suprapostural task. However, task accuracy in the suprapostural task was less affected by the focusing strategy. Moreover, with more attention directed to maintain postural balance, older adults and low back pain patients tended to reduce response time and increase suprapostural error, but young healthy subjects tended to maintain suprapostural accuracy by increasing RT [45,46]. Hence, the delayed RT observed in the young adults in this study might reflect a compensatory strategy to optimize the postural task [47]. In contrast, due to limited information-processing capacity, loss of precise control is over the speed in older adults, especially when producing a predefined amount of force in a short time [48–50]. In addition, based on our study design, participants received the visual feedback about force error but not force RT in the D_SF condition, and they did not receive any force-related feedback in the D_PF condition. It has been known that the absence of visual feedback would deteriorate the control of force accuracy, but not necessary affect the speed of force output [51,52]. Therefore, older adults with less available attentional resource might sacrifice the accuracy of force-matching task by arbitrarily executing force-matching with a fast RT when adopting a PF strategy in a dual-task condition. The distinct strategies employed by the young and old adults may be a manifestation of age-dependent reductions in residual resources for undertaking a postural-suprapostural task, especially when the postural task was prioritized. A positive posture ApEn DTE suggests that postural automaticity increased with addition of the force-matching task in young adults. The young adults were likely to engage extra resources in the execution of an added force-matching task. However, the capacity for resource expansion was limited in older adults, as indicated by a negative value for posture ApEn DTE. When the majority of attentional resources were directed to the postural task, resource competition between the postural and suprapostural tasks in older adults was more intense, resulting in a degradation of force-matching accuracy.

A finding of particular importance in this study is that SF strategy is a more appropriate strategy for a posture-motor dual-tasking in both healthy young and older adults than PF strategy, which is not in line with findings obtained in several posture-cognitive dual-tasking studies [17,18]. When focusing on a suprapostural task that demands physical precision (e.g., continuous texting or bimanual phone holding), postural stability could be improved due to a compensatory mechanism in the CNS that ensures stability, but this effect was not observed in a cognitive suprapostural task [53]. In contrast to cognitive tasks, posture kinematics is more susceptible to a motor task; therefore, CNS would voluntarily increase balance stability in order to avoid a deterioration of suprapostural performance caused by postural instability. Besides fine motor control, coordination of a motor suprapostural task and postural task is an executive function, which requires cognitive demands in prefrontal and frontal regions [54]. Thus, adding a force-matching task in an unstable posture was a complex posture-motor-cognitive task rather than a simple posture-motor task. Finally, postural control is a continuum ranging from “controlled to automatic” processing, depending on the level of postural demand and the capacity of attentional resources [43,55]. As postural difficulty increases, more controlled processing should be devoted to balance maintenance. The improved efficiency of dual-task performance resulting from an SF strategy might be affected when performing a suprapostural task with a highly attentionally demanding postural task, such as narrow walking or walking across obstacles. Because the variations in types of postural and suprapostural tasks contribute to the magnitude of dual-task interference [56], it is recommended that further studies use several combinations of postural and suprapostural tasks in the same experiment to explore multifaceted functions.

## Conclusions

In conclusion, SF strategy is the better movement strategy for both young and older adults during performance a motor suprapostural task while maintaining balance on an unstable surface. These findings of a potentially optimal task prioritization strategy may inform clinicians about the proper task prioritization instructions in fall prevention education or dual-task training. Because postural ability and attentional capacity might be more limited in patients with neurological diseases, such as stroke or Parkinson’s disease, compared to healthy older adults, future research should examine dual-task prioritization in neurologically impaired populations in order to develop more effective dual-task interventions.

## Supporting Information

S1 Dataset Raw Data. (XLSX)

## References

[1] Woollacott M, Shumway-Cook A. Attention and the control of posture and gait: A review of an emerging area of research. Gait Posture. 2002;16:1–14. 12127181 10.1016/s0966-6362(01)00156-4

[2] Huxhold O, Li SC, Schmiedek F, Lindenberger U. Dual-tasking postural control: Aging and the effects of cognitive demand in conjunction with focus of attention. Brain Res Bull. 2006;69:294–305. 10.1016/j.brainresbull.2006.01.002 16564425

[3] McNevin NH, Shea CH, Wulf G. Increasing the distance of an external focus of attention enhances learning. Psychol Res. 2003;67:22–29. 10.1007/s00426-002-0093-6 12589447

[4] Chen FC, Tsai CL. The mechanisms of the effect of light finger touch on postural control. Neurosci Lett. 2015;605:69–73. 10.1016/j.neulet.2015.08.016 26291485

[5] Polskaia N, Richer N, Dionne E, Lajoie Y. Continuous cognitive task promotes greater postural stability than an internal or external focus of attention. Gait Posture. 2015;41:454–458. 10.1016/j.gaitpost.2014.11.009 25554460

[6] Jehu DA, Desponts A, Paquet N, Lajoie Y. Prioritizing attention on a reaction time task improves postural control and reaction time. Int J Neurosci. 2015;125:100–106. 10.3109/00207454.2014.907573 24655152

[7] Horak FB, Shupert CL, Mirka A. Components of postural dyscontrol in the elderly: A review. Neurobiol Aging. 1989;10:727–738. 2697808 10.1016/0197-4580(89)90010-9

[8] Lacour M, Bernard-Demanze L, Dumitrescu M. Posture control, aging, and attention resources: Models and posture-analysis methods. Neurophysiol Clin. 2008;38:411–421. 10.1016/j.neucli.2008.09.005 19026961

[9] Woollacott MH. Editorial: Systems Contributing to Balance Disorders in Older Adults. J Gerontol A Biol Sci Med Sci. 2000;55:M424–M428. 10952363 10.1093/gerona/55.8.m424

[10] Yogev-Seligmann G, Hausdorff JM, Giladi N. Do we always prioritize balance when walking? Towards an integrated model of task prioritization. Mov Disord. 2012;27:765–770. 10.1002/mds.24963 22419512

[11] Lion A, Spada RS, Bosser G, Gauchard GC, Anello G, Bosco P, et al “Postural first” principle when balance is challenged in elderly people. Int J Neurosci. 2014;124:558–566. 10.3109/00207454.2013.864288 24205810

[12] Schaefer S. The ecological approach to cognitive–motor dual-tasking: findings on the effects of expertise and age. Front Psychol. 2014;5. 10.3389/fpsyg.2014.01167 PMC4196472 25352820

[13] Lindenberger U, Marsiske M, Baltes PB. Memorizing while walking: increase in dual-task costs from young adulthood to old age. Psychol Aging. 2000; 15:417–436. 11014706 10.1037//0882-7974.15.3.417

[14] Li KZ, Lindenberger U, Freund AM, Baltes PB. Walking while memorizing: age-related differences in compensatory behavior. Psychol Sci. 2001;12:230–237. 11437306 10.1111/1467-9280.00341

[15] Bernard-Demanze L, Dumitrescu M, Jimeno P, Borel L, Lacour M. Age-related changes in posture control are differentially affected by postural and cognitive task complexity. Curr Aging Sci. 2009;2:135–149. 20021408

[16] Berger L, Bernard-Demanze L. Age-related effects of a memorizing spatial task in the adults and elderly postural control. Gait Posture. 2011;33:300–302. 10.1016/j.gaitpost.2010.10.082 21087866

[17] Verghese J, Kuslansky G, Holtzer R, Katz M, Xue X, Buschke H, et al Walking While Talking: Effect of Task Prioritization in the Elderly. Arch Phys Med Rehabil. 2007;88:50–53. 10.1016/j.apmr.2006.10.007 17207675 PMC1894901

[18] Yogev-Seligmann G, Rotem-Galili Y, Mirelman A, Dickstein R, Giladi N, Hausdorff JM. How does explicit prioritization alter walking during dual-task performance? Effects of age and sex on gait speed and variability. Phys Ther. 2010;90:177–186. 10.2522/ptj.20090043 20023000 PMC2816029

[19] Deviterne D, Gauchard GC, Jamet M, Vançon G, Perrin PP. Added cognitive load through rotary auditory stimulation can improve the quality of postural control in the elderly. Brain Res Bull. 2005;64:487–492. 10.1016/j.brainresbull.2004.10.007 15639544

[20] Prado JM, Stoffregen TA, Duarte M. Postural Sway during Dual Tasks in Young and Elderly Adults. Gerontology. 2007;53:274–281. 10.1159/000102938 17510558

[21] Hartley AA. Age differences in dual-task interference are localized to response-generation processes. Psychol Aging. 2001;16:47–54. 11302367 10.1037/0882-7974.16.1.47

[22] Tsang PS. Ageing and attentional control. Q J Exp Psychol (Hove). 2013;66: 1517–1547. 23281799 10.1080/17470218.2012.752019 PMC4143253

[23] Laessoe U, Hoeck HC, Simonsen O, Voigt M. Residual attentional capacity amongst young and elderly during dual and triple task walking. Hum Mov Sci. 2008;27:496–512. 10.1016/j.humov.2007.12.001 18226839

[24] Siu KC, Woollacott MH. Attentional demands of postural control: The ability to selectively allocate information-processing resources. Gait Posture. 2007;25:121–126. 10.1016/j.gaitpost.2006.02.002 16554158

[25] Zaepffel M, Brochier T. Planning of visually guided reach-to-grasp movements: inference from reaction time and contingent negative variation (CNV). Psychophysiology. 2012;49:17–30. 10.1111/j.1469-8986.2011.01277.x 21895686

[26] Zaepffel M, Trachel R, Kilavik BE, Brochier T. Modulations of EEG beta power during planning and execution of grasping movements. PLoS One. 2013;8: e60060 10.1371/journal.pone.0060060 23555884 PMC3605373

[27] Navon D. Resources—a theoretical soup stone? Psychol Rev. 1984;91:216–234.

[28] Tsang PS, Velazquez VL, Vidulich MA. Viability of resource theories in explaining time-sharing performance. Acta Psychol (Amst). 1996;91:175–206. 8677806 10.1016/0001-6918(94)00055-7

[29] Pincus S. Approximate entropy (ApEn) as a complexity measure. Chaos. 1995;5: 110–117. 10.1063/1.166092 12780163

[30] Donker S, Roerdink M, Greven A, Beek P. Regularity of center-of-pressure trajectories depends on the amount of attention invested in postural control. Exp Brain Res. 2007;181:1–11. 10.1007/s00221-007-0905-4 17401553 PMC1914290

[31] Kuczyński M, Szymańska M, Bieć E. Dual-task effect on postural control in high-level competitive dancers. J Sports Sci. 2011;29:539–545. 10.1080/02640414.2010.544046 21294035

[32] Barrett G, Shibasaki H, Neshige R. A computer-assisted method for averaging movement-related cortical potentials with respect to EMG onset. Electroencephalogr Clin Neurophysiol. 1985;60:276–281. 2578938 10.1016/0013-4694(85)90042-2

[33] Tomberg C, Levarlet-Joye H, Desmedt JE. Reaction times recording methods: reliability and EMG analysis of patterns of motor commands. Electroencephalogr Clin Neurophysiol. 1991;81:269–278. 1714821 10.1016/0168-5597(91)90013-n

[34] Nordez A, Gallot T, Catheline S, Guével A, Cornu C, Hug F. Electromechanical delay revisited using very high frame rate ultrasound. J Appl Physiol (1985). 2009;106:1970–1975. 19359617 10.1152/japplphysiol.00221.2009

[35] Abellaneda S, Guissard N, Duchateau J. The relative lengthening of the myotendinous structures in the medial gastrocnemius during passive stretching differs among individuals. J Appl Physiol (1985). 2009;106:169–177. 18988765 10.1152/japplphysiol.90577.2008

[36] Mitra S. Adaptive utilization of optical variables during postural and suprapostural dual-task performance: Comment on Stoffregen, Smart, Bardy, and Pagulayan (1999). J Exp Psychol Hum Percept Perform. 2004;30:28–38. 10.1037/0096-1523.30.1.28 14769066

[37] Mitra S, Fraizer EV. Effects of explicit sway-minimization on postural–suprapostural dual-task performance. Hum Mov Sci. 2004;23:1–20. 10.1016/j.humov.2004.03.003 15201038

[38] Wulf G, McNevin N, Shea CH. The automaticity of complex motor skill learning as a function of attentional focus. Q J Exp Psychol A. 2001;54:1143–1154. 10.1080/713756012 11765737

[39] Borel L, Alescio-Lautier B. Posture and cognition in the elderly: interaction and contribution to the rehabilitation strategies. Neurophysiol Clin. 2014;44:95–107. 10.1016/j.neucli.2013.10.129 24502910

[40] Termoz N, Halliday SE, Winter DA, Frank JS, Patla AE, Prince F. The control of upright stance in young, elderly and persons with Parkinson's disease. Gait Posture. 2008;27:463–470. 10.1016/j.gaitpost.2007.05.015 17644337

[41] Zwergal A, Linn J, Xiong G, Brandt T, Strupp M, Jahn K. Aging of human supraspinal locomotor and postural control in fMRI. Neurobiol Aging. 2012;33:1073–1084. 10.1016/j.neurobiolaging.2010.09.022 21051105

[42] Van Impe A, Coxon JP, Goble DJ, Wenderoth N, Swinnen SP. Age-related changes in brain activation underlying single- and dual-task performance: Visuomanual drawing and mental arithmetic. Neuropsychologia. 2011;49:2400–2409. 10.1016/j.neuropsychologia.2011.04.016 21536055

[43] Boisgontier MP, Beets IAM, Duysens J, Nieuwboer A, Krampe RT, Swinnen SP. Age-related differences in attentional cost associated with postural dual tasks: Increased recruitment of generic cognitive resources in older adults. Neurosci Biobehav Rev. 2013;37:1824–1837. 10.1016/j.neubiorev.2013.07.014 23911924

[44] Kelly V, Janke A, Shumway-Cook A. Effects of instructed focus and task difficulty on concurrent walking and cognitive task performance in healthy young adults. Exp Brain Res. 2010;207:65–73. 10.1007/s00221-010-2429-6 20931180 PMC3058115

[45] Sherafat S, Salavati M, Takamjani IE, Akhbari B, Mohammadi Rad S, Mazaheri M, et al Effect of Dual-Tasking on Dynamic Postural Control in Individuals With and Without Nonspecific Low Back Pain. J Manipulative Physiol Ther. 2014;37:170–179. 10.1016/j.jmpt.2014.02.003 24636612

[46] Tomporowski PD, Audiffren M. Dual-task performance in young and older adults: speed-accuracy tradeoffs in choice responding while treadmill walking. J Aging Phys Act. 2014;22:557–563. 10.1123/japa.2012-0241 24306656

[47] Endrass T, Schreiber M, Kathmann N. Speeding up older adults: Age-effects on error processing in speed and accuracy conditions. Biol Psychol. 2012;89:426–432. 10.1016/j.biopsycho.2011.12.005 22197882

[48] Rabbitt P. How old and young subjects monitor and control responses for accuracy and speed. Br J Psychol. 1979;70:305–311.

[49] Sosnoff JJ, Newell KM. Aging, visual intermittency, and variability in isometric force output. J Gerontol B Psychol Sci Soc Sci. 2006;61:P117–124. 16497955 10.1093/geronb/61.2.p117

[50] Vieluf S, Godde B, Reuter EM, Voelcker-Rehage C. Age-related differences in finger force control are characterized by reduced force production. Exp Brain Res. 2013;224:107–117. 10.1007/s00221-012-3292-4 23076430

[51] Therrien AS, Balasubramaniam R. Timing and visual feedback constraints on repetitive finger force production. Exp Brain Res. 2010;201:673–679. 10.1007/s00221-009-2084-y 19936722

[52] Critchley K, Kokubu M, Iemitsu M, Fujita S, Isaka T. Age-related differences in the availability of visual feedback during bimanual pinch. Eur J Appl Physiol. 2014;114:1925–1932. 10.1007/s00421-014-2916-8 24907975

[53] Marone JR, Patel PB, Hurt CP, Grabiner MD. Frontal plane margin of stability is increased during texting while walking. Gait Posture. 2014;40:243–246. 10.1016/j.gaitpost.2014.04.188 24798610

[54] Beurskens R, Steinberg F, Antoniewicz F, Wolff W, Granacher U. Neural Correlates of Dual-Task Walking: Effects of Cognitive versus Motor Interference in Young Adults. Neural Plast. 2016;2016:8032180 10.1155/2016/8032180 27200192 PMC4855015

[55] Stins JF, Michielsen ME, Roerdink M, Beek PJ. Sway regularity reflects attentional involvement in postural control: Effects of expertise, vision and cognition. Gait Posture. 2009;30:106–109. 10.1016/j.gaitpost.2009.04.001 19411174

[56] Bock O. Dual-task costs while walking increase in old age for some, but not for other tasks: an experimental study of healthy young and elderly persons. J Neuroeng Rehabil. 2008;5:27 10.1186/1743-0003-5-27 19014544 PMC2596160

